# Incorporating CNN Features for Optimizing Performance of Ensemble Classifier for Cardiovascular Disease Prediction

**DOI:** 10.3390/diagnostics12061474

**Published:** 2022-06-15

**Authors:** Furqan Rustam, Abid Ishaq, Kashif Munir, Mubarak Almutairi, Naila Aslam, Imran Ashraf

**Affiliations:** 1 Department of Software Engineering, School of Systems and Technology, University of Management and Technology, Lahore 54770, Pakistan; furqan.rustam1@gmail.com; 2 Department of Computer Science, Khwaja Fareed University of Engineering and Information Technology, Rahim Yar Khan 64200, Pakistan; airgcu@gmail.com; 3 Department of Information Technology, Khwaja Fareed University of Engineering and Information Technology, Rahim Yar Khan 64200, Pakistan; kashif.munir@kfueit.edu.pk; 4 College of Computer Science and Engineering, University of Hafr Albatin, Hafr Al-Batin 39524, Saudi Arabia; 5 Department of Computer Science, School of Systems and Technology, University of Management and Technology, Lahore 54770, Pakistan; nailaaslam2020@yahoo.com; 6 Department of Information and Communication Engineering, Yeungnam University, Gyeongsan 38541, Korea

**Keywords:** cardiovascular disease prediction, transfer learning, feature extraction, deep learning

## Abstract

Cardiovascular diseases (CVDs) have been regarded as the leading cause of death with 32% of the total deaths around the world. Owing to the large number of symptoms related to age, gender, demographics, and ethnicity, diagnosing CVDs is a challenging and complex task. Furthermore, the lack of experienced staff and medical experts, and the non-availability of appropriate testing equipment put the lives of millions of people at risk, especially in under-developed and developing countries. Electronic health records (EHRs) have been utilized for diagnosing several diseases recently and show the potential for CVDs diagnosis as well. However, the accuracy and efficacy of EHRs-based CVD diagnosis are limited by the lack of an appropriate feature set. Often, the feature set is very small and unable to provide enough features for machine learning models to obtain a good fit. This study solves this problem by proposing the novel use of feature extraction from a convolutional neural network (CNN). An ensemble model is designed where a CNN model is used to enlarge the feature set to train linear models including stochastic gradient descent classifier, logistic regression, and support vector machine that comprise the soft-voting based ensemble model. Extensive experiments are performed to analyze the performance of different ratios of feature sets to the training dataset. Performance analysis is carried out using four different datasets and results are compared with recent approaches used for CVDs. Results show the superior performance of the proposed model with 0.93 accuracy, and 0.92 scores each for precision, recall, and F1 score. Results indicate both the superiority of the proposed approach, as well as the generalization of the ensemble model using multiple datasets.

## 1. Introduction

Cardiovascular disease (CVD) has been regarded as the leading cause of death around the world [1]. Reports show that a person dies every 36 s in the United States (US) from CVDs implying one CVD death in every four deaths [2,3]. Worse still, 32% of the total deaths in the world are due to CVDs indicating that one death in every three deaths is caused by heart-related disorders [4]. The European cardiology society (ESC) statistics show that approximately 26 million adults around the world are diagnosed with CVD. About half of the patients diagnosed with CVD die in one to two years. The high mortality rate from CVDs leads to a significant burden on the health care system. About 3% of the total budget of the health care system is used for CVD treatment [5]. In the US alone, approximately 291 billion dollars are spent from 2014 to 2015 on the health care system and medicine [6].

WHO statistics indicate that CVD is the leading cause of death in the world [7], with approximately 17.9 million deaths around the world with high mortality in Asian and low to middle-income countries [8]. Males are most likely to be affected by CVD as compared to females, especially in the middle or old age [9,10]. Due to many contributing factors leading to different symptoms and different levels of symptoms concerning age, demographics, and ethnicity, CVDs are difficult to diagnose. High blood pressure, abnormal pulse rate, cholesterol levels, etc. contribute to different types of CVD [11]. CVD symptoms might be different in different genders; for example, a male might have chest discomfort such as extreme fatigue, nausea, and shortness of breath while females might have just chest pain [3]. These factors make the diagnosis process challenging and demanding. Early diagnosis of CVD can be very difficult [12]. For diagnosing CVD, multiple tests are required, in addition to trained and expert staff. A lack of staff and medical experts can lead to a large number of false predictions with the potential risk of mortality. Angiography is considered the most accurate and precise method for the prediction of CVD, but its high cost makes it less accessible, especially in low-income countries [13].

Surgical treatment of CVD is a challenging task, especially in developing countries due to the non-availability of trained staff, testing equipment, and other resources [14]. Physical examinations, despite being expensive, are not free from flaws and imperfections. Recently, electronic health records (EHRs) have exhibited a substantial potential to offer useful insights for research in clinical medicine. A large body of work has been presented using EHRs for CVD as well and can be very helpful for the early diagnosis of CVD. Both machine learning and deep learning models have been deployed for CVD prediction; however, they have several limitations. For example, the provided prediction accuracy and F measures are not high enough. Similarly, the results are not generalizable, as using a different dataset might show very different results concerning true positive rates. The last but most important problem is feature engineering which has a direct influence on the performance of prediction models. Predominantly, the EHRs have a low number of features and a good fit of the model is not achieved which leads to poor prediction performance. This study resolves these problems by proposing the use of features extracted by a convolutional neural network (CNN) with machine learning models. In brief, this study makes the following contributions:

- A novel method of using CNN with machine learning models is developed, where CNN is used to enlarge the feature set and linear models are used for predicting CVD. Extensive experiments are performed to evaluate the impact of the ratio of an enlarged feature set on the prediction performance.
- A soft-voting-based ensemble model is introduced that leverages three linear models including stochastic gradient descent classifier (SGDC), logistic regression (LR), and support vector machine (SVM) to be trained on a CNN features set.
- Performance evaluation is carried out on four different datasets for results generalization using accuracy, precision, recall, and F1 score. Performance analysis is realized with several state-of-the-art approaches, and deep learning gated recurrent unit (GRU) and CNN models are also implemented. Results show significant improvement in CVD prediction.

This paper is further organized into four sections. Starting with the related work in Section 2, it discusses the proposed approach, datasets, and models in Section 3. Results and discussions are given in Section 4 while the conclusions are provided in Section 5.

## 2. Related Work

Keeping in view the importance of CVD diagnosis, several approaches have been presented on the topic. Predominantly, such approaches are based on machine and deep learning models that utilize two publicly available datasets including ‘Statlog’ and ‘Cleveland’ for CVDs. This section discusses the most recent and relevant works that offer high prediction accuracy.

Reddy et al. [15] proposed a machine learning-based system comprising attribute evaluators. The authors use ten different machine learning models from different categories, such as Bayesian-based models, tree-based models, rules-based models, etc. To obtain high accuracy for heart disease prediction, they use all attributes of the Cleveland dataset, as well as the optimal attributes obtained from the three attribute evaluators. Results show that sequential minimal optimization obtains an accuracy of 85.148% using the full set of attributes and 86.468% accuracy using optimal attributes. Similarly, a heart disease prediction model is developed by the Perumal et al. [16], which uses the Cleveland dataset. The study uses feature standardization and feature reduction using principal component analysis for training the models. Findings indicate that accuracy scores of 87% and 85% can be accomplished with LR and SVM, respectively.

Along the same lines, Ref. [17] makes use of five different machine learning models such as SVM, KNN, RF, decision tree (DT), and LR on the UCI 303 records dataset that has 10 attributes. Using a standard training and testing procedure, a maximum accuracy of 85.71% with receiver operating characteristic curve-area under the curve (ROC-AUC) of 0.8675 is obtained using a fine-tuned RF. In the same way, Ref. [18] presents a machine learning-based classification model for CVD prediction using the Cleveland dataset. For training, the feature reduction technique is used to select the 4 highly contributing attributes out of 13 attributes. The highest accuracy is obtained by both LR and Gaussian Naive Bayes (GNB) each with an 82.75% accuracy and 0.87 AUC. Shah et al. present a supervised learning algorithm-based model in [19] with the Cleveland dataset. The Cleveland dataset contains 303 instances and 76 attributes, out of which only 14 attributes are used by this study. Results suggest that the maximum accuracy of 90.789% is obtained using KNN. Similarly, study [20] used four different benchmark datasets to perform CVD prediction. The performance is analyzed using the top 2 and top 4 features/attributes from the datasets. Ten machine learning classifiers show appropriate classification and predictive performance for CVDs using the top two attributes. Similarly, three main attributes for the detection and prediction of CVDs are identified. Results show that accuracy scores of 81.32% and 77.84% are obtained on the Cleveland and Framingham dataset, respectively, while 74.44% and 73.38% accuracy scores are obtained on the Faisalabad institute of cardiology and South African health datasets, respectively.

Amin et al. [21] sought to identify the key features and data mining techniques for enhancing the accuracy of heart disease prediction. A series of predictive models are developed using different combinations of attributes and seven machine learning models. The results reflect that the best-performing model achieves an accuracy of 87.4%. Mohan et al. [22] propose a machine learning-based heart disease prediction model (HRFLM) that combines a linear model with features obtained from RF. HRFLM operates with diverse feature configurations and various classification techniques. Experimental results indicate that the highest accuracy of 88.7% is obtained using the proposed HRFLM. Similarly, Ref. [23] uses six machine learning techniques for heart disease prediction. The maximum accuracy of 85% is obtained using LR on the Statlog dataset. Both individual and ensemble learning approaches such as J48, MLP, Bayes Net, NB, RF, and random tree (RT) are investigated for CVD prediction in [24]. RT proved to be highly accurate with an accuracy of 70.77%. The study subsequently employed a new-fangled method that achieved an accuracy of 80%.

Several points can be derived from the above-discussed research works. First, Statlog and Cleveland datasets are the most used datasets for CVD prediction. Secondly, despite the high accuracy of deep learning in general, predominantly machine learning models SVM, KNN, RF, DT, LR, etc. are used for CVD prediction. Primarily, the availability of small-sized datasets makes machine learning models appropriate and more suitable for the task. Finally, the provided accuracy for CVD prediction requires further research and investigation to enhance the performance of machine learning models. This study contributes to achieving this goal by proposing the use of CNN features to enrich the feature set for obtaining higher prediction accuracy.

## 3. Methods

### 3.1. Proposed Methodology

**Figure 1:**
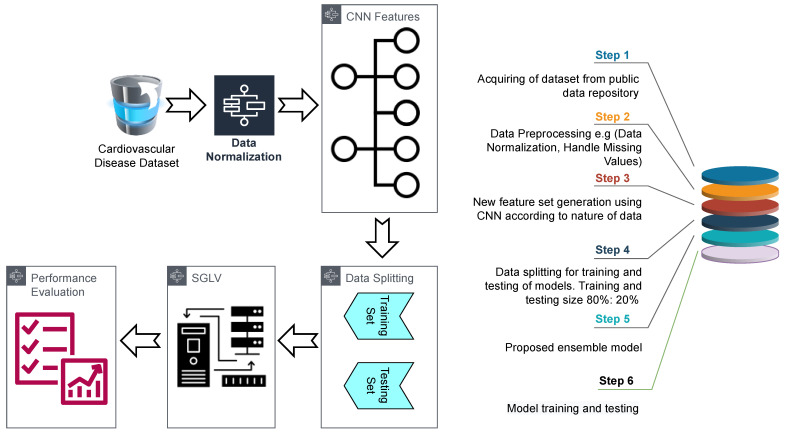
The architecture of the proposed methodology.

Taking the performance of machine learning models into consideration, this study leverages supervised machine learning models for CVD prediction. The proposed methodology is shown in Figure 1. It follows a sequence of operations, starting with the dataset collection. This research uses a publicly available dataset ‘Heart Failure Prediction Dataset (HFPD)’ from Kaggle [25]. The dataset is developed by combining five datasets with 11 common features, which makes it the largest available dataset for heart disease so far.

The dataset contains a total of 918 observations and has two classes. It comprises 11 attributes that are grouped under different types, such as numeric, nominal, binary, etc. A brief description of each attribute is provided in Table 1.

The dataset used for experiments contains numerical values and categorical features where categorical features indicate the string data. For example, the ’sex’ attribute in Table 1 has categorical values and contains ’M’ and ’F’ for males and females, respectively. Categorical values are easily understood by humans; however, such values are not fed into machine learning models for training. Conversion or data normalization aims at transforming these values into numeric values which are appropriate for machine learning models. For converting categorical values to numeric values, two methods can be used: dummy variable encoding and label encoding. Dummy variable encoding uses 0 and 1 to indicate the exclusion or inclusion of a category, irrespective of the number of categories. This study uses label encoding for transforming categorical data into numeric data which makes the data deployable with the machine learning and deep learning models directly. We deployed the encoding technique on the whole dataset using the Panda library of Python, and the results are shown in Table 2.

After data normalization, data are split into training and testing sets with a ratio of 0.8 to 0.2 for training and testing of models, respectively. The split ratio is defined empirically based on the performance evaluations made using different split ratios of 0.75:0.25, 0.70:0.30, and 0.85:0.15. The best results are obtained with a 0.80:0.20 train-test split ratio. The number of samples for training and testing is given in Table 3. Class 0 indicates the healthy people while Class 1 represents CVD patients.

### 3.2. Proposed Ensemble Model Convolutional SGLV

Predominantly, CVD datasets have a small number of features that is not appropriate to obtain higher performance using the machine learning algorithms. Often, linear models such as LR, SGDC, and SVM show good performance with a large feature set [26,27]. To resolve these issues, this study proposes an approach called ConvSGLV. The ConvSGLV is the combination of deep learning and machine learning models. Initially, it uses the CNN model for feature engineering to increase the size of the feature set. Later, the enriched feature set is used with the ensemble model that combines three linear models including SGD, LR, and SVM to make the final prediction using a soft voting criterion.

#### 3.2.1. Convolutional Neural Networks

The CNN model for feature engineering consists of four layers including the embedding layer, 1D convolutional layer, max-pooling layer, and flatten layer. The embedding layer takes 11 features of the CVD dataset with 20,000 vocabulary size and 300 output dimensions with an input length of 11. The embedding layer is followed by the 1D convolutional layer with 5000 filters, 2 × 2 kernel size, and the rectifier linear unit (ReLU) activation function. To map the important features from 1D convolution output, a max-pooling layer with a 2 × 2 pool size is used. In the end, a Flatten layer is incorporated to convert the output again into a one-dimensional array for machine learning models [28].

Suppose that the CVD dataset *X* consists of a tuple set $\left(fs_{i},tc_{i}\right)$, where *i* is the index of the tuple, fs is the feature set, and tc is the target class column; then, the embedding layer can be used to convert the training set into the required input format as

(1) $$\begin{array}{c} EL=embedding\_layer(V_{s},O_{s},I)\\ EO_{s}=EL\left(fs\right) \end{array}$$

where $EO_{s}$ represents the output of the embedding layer which generates the input for the convolutional layer, and EL is the embedding layer that consists of three parameters; one vocabulary size $V_{s}$, two output dimensions, and three input lengths.

Vocabulary size defines how much bigger the input can be in the model. The vocabulary size is set to 20,000, indicating that the model can take inputs within the range of 0 to 20,000. Second, we used the output dimension parameter $O_{s}$ with the value 300, which indicates that, when the input data passes through the embedding layer, its output dimension is 300. The third parameter is the input length *I*, which represents the number of features in the original dataset which are 11. The embedding layer processes the input data and generates the output for further processing by the CNN model. The output dimensions of the embedding layer are $EO_{s}=\left(None,11,300\right)$:

(2) $$1D-Conv_{s}=CNN\left(F,K_{s},AF\right)\leftarrow EO_{s}$$

where $1D-Conv_{s}$ is the output by the 1D convolutional layer.

The $1D-Conv_{s}$ is extracted from the output of the embedding layer. We used 5000 filters, i.e., F=5000, in CNN layers and a kernel size of $K_{s}=2\times 2$. A rectified linear unit (ReLU) activation function AF is used to set all negative values to zero in the $1D-Conv_{s}$ output matrix while others remain constant:

(3) $$f\left(x\right)=max{\left(0,E\right)}_{s}$$

The max-pooling layer is used to map the significant features from the CNN. A pool size of 2×2 is used for the feature map. Here, $F_{map}$ are features after max-pooling, $P_{s}=2$ is the pooling window size, and S−2 is the stride:

(4) $$C_{f}=F_{map}=\lfloor \frac{I-P_{s}}{S}\rfloor +1$$

In the end, a Flatten layer is used to convert 3D data into 1D because machine learning models work on 1D data. In this way, a total of 25,000 features are obtained to train machine learning models. The below equation shows the features for the training of learning models:

$$C_{f}=(\left[\begin{array}{ccc} 1,1 & \cdots & 1,14\\ \vdots & \ddots & \vdots \\ 918,1 & \cdots & 918,14 \end{array}\right])=\left[\begin{array}{ccc} 1,1 & \cdots & 1,25,000\\ \vdots & \ddots & \vdots \\ 918,1 & \cdots & 918,25,000 \end{array}\right]$$

#### 3.2.2. Stochastic Gradient Logistic Vector

This study proposes an ensemble model which is a combination of three linear models LR, SVM, and SGDC. These models are used for ensemble because of their best individual performance on the experimental dataset. Additionally, these models perform well on a large feature set data with the binary class problem [29,30]. The models are joined using the majority voting criteria.

LR is a statistical model used for the classification problem especially and can perform significantly better for binary classification. LR uses a logistic function to solve the classification problem and can be defined as

(5) $$f\left(x\right)=\frac{1}{1+e^{-x}}$$

The linear regression equation modifies the logistic function to adopt the model for binary classification and provides the between 0 and 1. The following equation shows the hypothesis of LR

(6) $$z=\beta_{0}+\beta_{1}X$$

(7) $$h\theta \left(x\right)=\frac{1}{1+e^{-(\beta_{0}+\beta_{1}X)}}$$

The second model in the SGLV model is SVM, which is a linear model used for classification and regression problems. SVM uses hyper-planes to classify the data and is easy to deploy, especially for binary classification problems. SVM draws multiple hyperplanes to separate the data and selects the one that classifies the data with a high-class boundary margin. The hyperplane can be defined as

(8) $$W^{T}x+b=0$$

where *w* is weight vector, *x* is input vector, and *b* is bias.

The best hyperplane can give significant results on linear data and binary classification problems. We can also write it as

(9) $$W^{T}x_{i}+b=\left[\begin{array}{c} +1\\ 0\\ -1 \end{array}\right]$$

**Figure 2:**
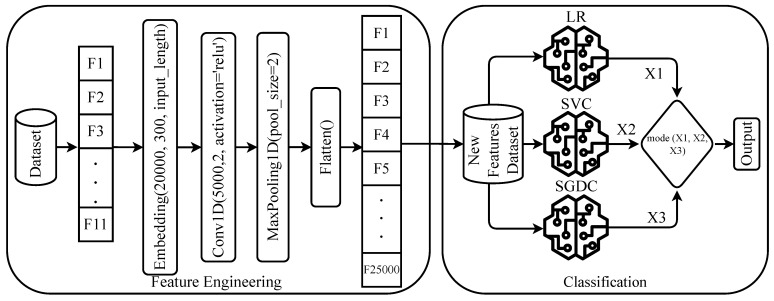
Architecture of the proposed ConvSGLV.

The third model in SGVL is SGDC, which is also a linear model and is used with an SGD optimizer. SGD randomly picks one data point from the whole data set at each iteration to reduce the computational complexity. Three linear models are used with the best hyperparameters setting found using the grid search method and combined under majority voting criteria. The SGLV is defined as

(10) $$\begin{array}{c} LR_{t}=LR_{train}\left(C_{f}\right) \end{array}$$

(11) $$\begin{array}{c} SVM_{t}=SEM_{train}\left(C_{f}\right) \end{array}$$

(12) $$\begin{array}{c} SGDC_{t}=SGCD_{train}\left(C_{f}\right) \end{array}$$

where $LR_{t},\;SVM_{t},\;\mathrm{and}\;SGDC_{t}$ represent the trained models on CNN features and the predictions of all the trained models on test data can be defined as

(13) $$p_{1},p_{2},p_{3}=LR_{t}\left(test\right),SVM_{t}\left(test\right),SGDC_{t}\left(test\right)$$

where $p_{1}$, $p_{2}$, and $p_{3}$ are the predictions by individual models and majority voting on models’ prediction can be defined as

(14) $$SGLV_{p}=mode\left\{p_{1},p_{2},p_{3}\right\}$$

where $SGLV_{p}$ is the final prediction of SGLV models using the majority voting criteria and the approach diagram is shown in Figure 2.

### 3.3. Evaluation

For performance evaluation, several evaluation parameters are used such as accuracy, precision, recall, and F1 score. These evaluation parameters can be calculated using the confusion matrix values including true positive (TP), true negative (TN), false positive (FP), and false negative (FN):

- TP is when the model predicts the record as 0 and the actual label of the record is also 0;
- TN is when the model predicts a record as 1 and the actual label of the record is also 1;
- FP is when the model predicts the record as 1 and the actual label of the record is 0;
- FN is when the model predicts the record as 0 and the actual label of the record is 1.

Equations used to calculate accuracy, precision, recall, and F1 score are provided as follows:

(15) $$\begin{array}{c} Accuracy=\frac{TP+TN}{TP+TN+FP+FN} \end{array}$$

(16) $$\begin{array}{c} Precision=\frac{TP}{TP+FP} \end{array}$$

(17) $$\begin{array}{c} Recall=\frac{TP}{TP+FN} \end{array}$$

(18) $$\begin{array}{c} F1\;score=2\times \frac{Precision\times Recall}{Precision+Recall} \end{array}$$

## 4. Results

The study performs several experiments to analyze the performance of the proposed approach in comparison to other machine learning and deep learning models. All experiments are performed on the Intel Corei7 7th generation machine with Windows 10 operating system. TensorFlow, Keras, and Sci-kit learn frameworks are used in Python language to implement the proposed approach and machine learning and deep learning models. Experiments are performed separately using the original feature set from the heart disease dataset, as well as the CNN features.

### 4.1. Performance of Models Using Original Features

Initially, the experiments are performed using the original feature set from the heart disease dataset, and the results are provided in Table 4. Results indicate that the tree-based ensemble model extra tree classifier (ETC) outperforms other models with a significant accuracy of 0.88. Similarly, other tree-based models RF and AdaBoost (ADA) classifier also achieve good accuracy scores of 0.85 and 0.83, respectively. Linear models such as LR and SVM show better performance in terms of accuracy score each with 0.86 accuracy. However, the ensemble of the linear model shows better performance as compared to individual linear models with a 0.87 accuracy score.

The performance of the tree-based model ETC is good as compared to linear models because often linear models work well with a large feature set. The tree-based models, on the other hand, can be good even on a small feature set. Despite the better performance of ETC, the accuracy does not meet the standards of heart prediction accuracy and must be further improved. For this purpose, further experiments are performed using CNN extracted features and an ensemble machine learning model.

### 4.2. Performance of Models Using CNN Features

The second set of experiments is carried out using the CNN features to analyze the performance of machine learning and the proposed ensemble model, and the results are shown in Table 5. The objective of using the features from the CNN model is to enlarge the feature set that is expected to increase the accuracy of linear models. Machine learning models are trained and tested using the CNN extracted features for these experiments.

Experiment results show that the proposed linear ensemble model SGLV shows significantly better results with the highest accuracy of 0.92 and outperforms all other models. It indicates a substantial improvement in the performance of SGLV and shows an improvement of 5.74% in the performance than its performance with the original features. In the same fashion, individual linear models also obtain better results with CNN features as compared to the original feature set. LR achieves a 0.90 accuracy while SVM and SGDC each obtain a 0.88 accuracy score indicating improvements of 0.04, and 0.02, respectively. Tree-based RF is also good with a 0.89 accuracy score; however, DT is the worst with a 0.79 accuracy score. This significant improvement in the accuracy of models is attributed to the increase in the number of features when CNN is used for feature extraction. Features generated from the CNN model are highly correlated with the target class and make data linearly separable, so linear models show better performance than other models.

### 4.3. Performance Comparison with Original vs. CNN Features

**Figure 3:**
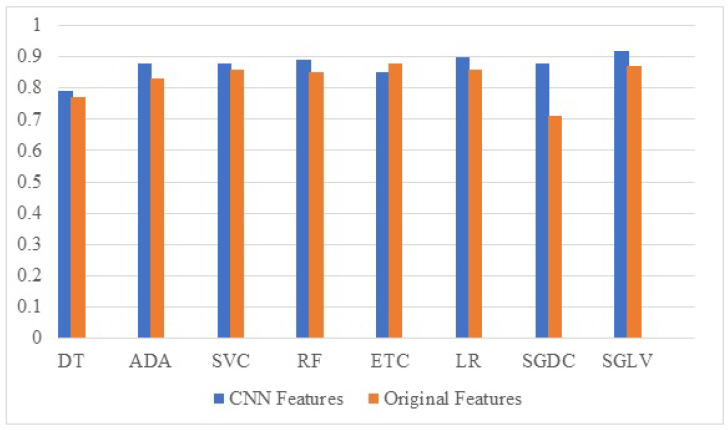
Comparison between model performance with proposed features and original features.

A performance comparison of the models’ results is carried out to show the change in the performance when using the CNN features. Comparison results are given in Figure 3. It shows that the performance of all the used models is elevated significantly when used with the proposed CNN features. Primarily, the large feature set generated from the CNN model provides better and more important features for the machine learning models, especially linear models to obtain a good fit which leads to improved results. Secondly, it is also possible that CNN features make the feature space more discriminative and linearly separable which helps linear models more than tree-based models. To corroborate this hypothesis, both feature sets are further analyzed.

**Figure 4:**
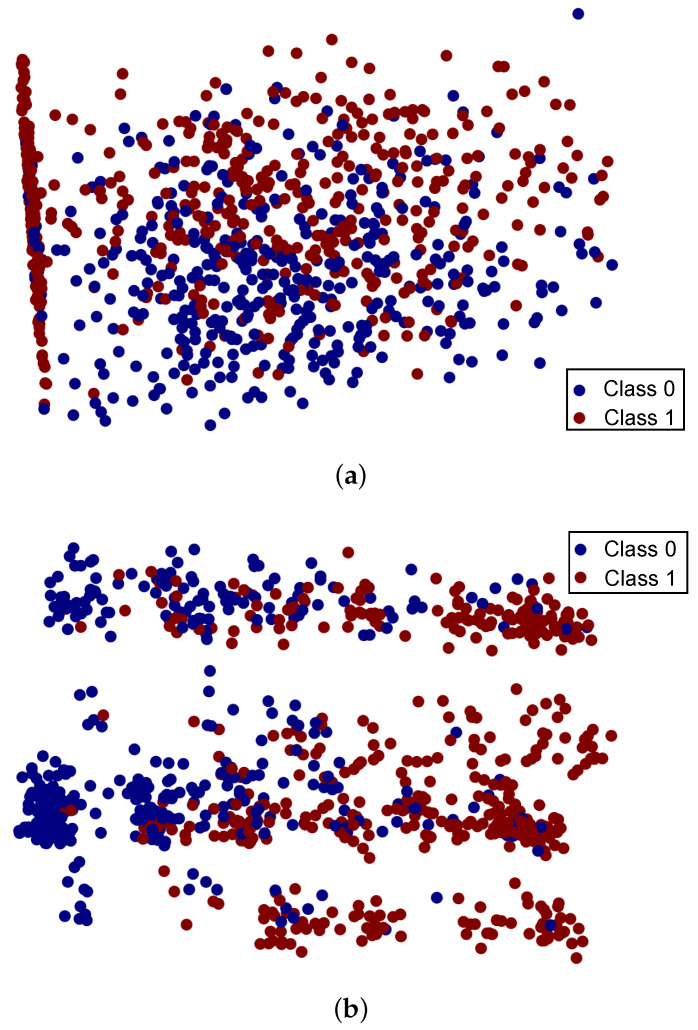
Visualization of the features sets, (**a**) original features and (**b**) CNN features set.

Figure 4 shows the plotted feature sets, both the original features and the CNN extracted features. It can be seen in Figure 4a that the original feature set does not have a discriminating distribution and features for both the classes occupy almost the same feature space. Consequently, the classification accuracy of both tree-based models and linear models is not very high with the original feature set. However, when the CNN model is used to extract the features, it has a twofold impact on the features. First, the feature set becomes large, as 25,000 features are extracted using CNN, which increases the number of features than the original dataset. Secondly, it increases the distance between the features of each class and makes them more separable. Figure 4 shows the feature space concerning the target classes for both the original 11 features dataset and 25,000 features dataset. We used PCA to visualize the feature space impact with the original dataset, as well as for CNN feature extraction. A scatter plot is used to visualize the feature space which takes two variables as input, but our datasets consist of 12 features and 25,000 features. Thus, we reduce the features into two variables using the PCA technique. PCA finds the maximum variance direction in high dimensional data and converts it into low dimension feature space compared to the original one. We apply first the data normalization technique and then the PCA technique to visualize the feature space. It can be observed that the samples for both the target classes are highly overlapped with the original feature set, while with augmented 25,000 features, samples are linearly separable, which helps to boost the accuracy of the models.

### 4.4. Influence of Number of Features

Further experiments are performed to define the role of the number of features as different sizes of the feature set may show very different results. For the most part, a large feature set is good for linear machine learning models to obtain a good fit. However, increasing the size of the feature set after a certain point would not yield any improvements. Instead, it may demote the performance and increase the computational complexity of the models. For this purpose, experiments are performed using 10,000, 15,000, 20,000, and 25,000 CNN features for all the models, and results are provided in Table 6. Experimental results suggest that the highest accuracy is obtained using a feature set of 25,000 features for the given dataset. The feature set size may vary concerning the nature of the dataset, the number of samples in the dataset, the number of classes, etc.

A comparison of all the models with respect to the number of correct predictions (CP) and the number of wrong predictions (WP), using both the original features and CNN features, is provided in Table 7. It shows that the highest performance for CVD prediction is by the proposed ensemble model SGLV with 170 CPs, followed by LR and RF with 166 and 164 CPs, respectively. This performance is significantly higher as compared to their performance using the original features where the highest CPs by the SGLV is 160 only. Results corroborate that enlarging the feature set using the CNN features improves the performance of machine learning models.

### 4.5. Validating the Performance of Proposed CNN Features

This study uses several datasets to validate the performance of the proposed idea of using the CNN features to enlarge the feature set for elevating the classification accuracy of the models. For this purpose, four additional datasets have been selected including Cleveland Heart Disease (CHD), Statlog Heart Disease (SHD), South African Heart Disease Dataset (SAHDD), and HFPD. The same procedure is followed, as described previously, to generate CNN features, and the results are presented in Table 8. Results confirm that using the CNN extracted features helps the machine learning models obtain improved performance.

### 4.6. Experimental Results of Deep Learning Models

Besides the machine learning models and the proposed ensemble model, two deep learning models are also implemented for the heart disease prediction task including CNN and GRU. The architecture of these models is optimized to achieve better accuracy; details related to the architecture of each model are given in Table 9. The models are compiled with binary_crossentropy loss function because of the binary target class and the ’Adam’ optimizer is used for optimization.

The models are trained using 100 epochs. Experimental results are provided in Table 10, which indicates that the CNN model shows comparatively better results than the GRU with an accuracy of 0.82. Despite that, the performance of deep learning models is inferior to machine learning models. Deep learning models are data-intensive and require a large number of samples for obtaining a good fit. The heart disease dataset is small and has only 11 features, which make it inappropriate for the deep learning models. The dataset is equally inappropriate for the machine learning models; however, the novel method of enlarging the feature elevates the performance of the machine learning models.

### 4.7. Computational Complexity of Models

We find the computational complexity of all models in terms of execution time for CVD prediction, and the results are given in Table 11. As expected, models take more time with 25,000 CNN features as compared to original features. Proposed SGLV takes 7.99 s for training and testing when used with 11 features; however, it is increased to 17.06 s when CNN features are used. An increase in the size of the feature set also increases the computational cost of other machine learning models. However, accuracy is more important as compared to the computational cost in the medical field. Thus, a trade-off can be made between the accuracy and the computational cost of the proposed approach.

### 4.8. Performance Comparison with State-of-the-Art Studies

A performance comparison is done for the proposed method to show its significance over recent heart disease prediction methods. For this purpose, results from the proposed approach are compared with state-of-the-art studies.

For comparison, three famous heart disease datasets are selected including the CHD, SHD, and SAHDD datasets. For a fair comparison, only those studies that use the same datasets for experiments have been selected. For the CHD dataset, Refs. [22,31,32,33,34] are selected. The study [22] proposed a hybrid RF with a linear model (HRFLM) for heart disease prediction. Ref. [31] used the machine learning model LR to achieve high accuracy results on the CHD dataset. Similarly, Ref. [32] used CHD to experiment with heart disease detection. The authors deployed Bernoulli Naive Bayes (BNB) and RF for prediction. BNB outperforms with an accuracy of 0.85. The study [33] used cluster-based DT learning (CDTL) and RF to obtain higher prediction accuracy. Another study [34] used ModifiedBoostARoota-CatBoost (MBAR-CB), CatBoost classifiers (CBC), ModifiedBoostARoota-XGBoost (MBAR-XGB), XGBoost Classifier (XGBC) for CHD, SHD, and SAHDD, respectively. The study [35] proposed an ensemble learning approach for heart disease prediction. The authors conducted experiments on the SHD and achieved an 0.88 accuracy score using the proposed ensemble model. A few of these studies perform experiments using more than one dataset. Table 12 shows the comparison between the proposed approach and these studies.

Table 12 shows that the proposed approach ConvSGLV outperforms the state-of-the-art approaches for heart disease prediction and shows significantly better performance. For example, the highest accuracy from state-of-the-art approaches on the CHD data are by [34] that obtains a 0.918 accuracy score; however, the proposed ConvSGLV obtains a 0.93 accuracy score. Similarly, using other datasets such as SHD and SAHDD, the performance of the ConvSGLV is superior to state-of-the-art approaches.

## 5. Conclusions

Cardiovascular diseases are becoming widespread with a dangerously higher mortality rate and current CVD prediction approaches lack high accuracy and robustness. Accurate and efficient CVD prediction necessitates intuitive and novel approaches to provide higher classification performance, which is not achievable with current methods and low attributed CVD datasets. This study proposes the novel use of a CNN enlarged feature set to overcome this limitation and proposes a linear ensemble model that is used with CNN features to obtain higher prediction performance. Extensive experiments are performed to validate the performance of the proposed approach with different machine learning and deep learning models using a different number of features from the CNN model. The findings suggest that the CNN feature set is large and more separable than the original feature set which helps to elevate the performance of the models. For performance validation, four datasets are used and performance comparison is conducted with the state-of-the-art approaches, which proves the supremacy of the proposed ConvSGLV model. The ConvSGLV outperforms all the models. Keeping in view the experiments with four different datasets, the performance of the ConvSGLV is robust and efficient. Results indicate that the performance of the proposed approach, although better than [34], is lower with the SAHDD dataset as compared to other datasets. It indicates the limitation on part of generalizing the results and requires further investigation. SAHDD dataset is imbalanced, has a small feature set than other datasets and fewer number of samples, which requires in-depth study using the proposed approach. Similarly, the computational complexity of the proposed is higher and a trade-off is needed between accuracy and computational complexity. In the future, we want to extend the proposed approach for the prediction of other diseases with a mixed feature set. We also intend to reduce the computational complexity of the approach without compromising the accuracy.

## Figures and Tables

**Table 1:** Description of dataset attributes.

Attribute	Data Type	Description
Age	Integer	This attribute contains the age of a patient (years).
Sex	String	This attribute contains the sex/gender of the patient in string format, [M = male, F = female]
Chest pain type	String	This attribute contains the type of the chest pain experienced by the patient in string format, [TA = typical angina, ATA = Atypical angina, NAP = Non-anginal pain, Asy = asymtomatic]
Resting BP	Integer	Resting blood pressure of the patient in mmHg
Cholesterol	Integer	Serum cholesterol in mm/dL
Fasting BS	Integer	Fasting blood sugar [1 = if fasting BS > 120 mg/dL, 0 = otherwise]
Resting ECG	String	Electrocardiogram (ECG) result [Normal = Normal, ST = having ST-T wave abnormality (T wave inversions and/or ST elevation or depression of >0.05 mV), LVH = showing probable or definite left ventricular hypertrophy by Estes’ criteria]
Max HR	Integer	This attribute contains the maximum heart rate of a patient [Numeric value between 60 and 202]
Exercise Angina	String	Exercise-induced angina [Y = yes, N = no]
Old Peak	Float	ST depression induced by exercise relative to rest
ST-Slope	String	Slop or the peak exercise ST segment [Up = upsloping, Flat = flat, Down = downsloping]
Heart Disease	Integer	Binary Target, [Class 1 = heart disease, Class 0 = normal]

**Table 2:** Results of the encoding technique on the sample dataset.

Age	Sex	ChestPainType	RestingBP	Choles.	FastingBS	RestingECG	MaxHR	Exer.Angina	Oldpeak	ST_Slope	HeartDisease
Before Encoding											
40	M	ATA	140	289	0	Normal	172	N	0.0	Up	0
49	F	NAP	160	180	0	Normal	156	N	1.0	Flat	1
37	M	ATA	130	283	0	ST	98	N	0.0	Up	0
After Encoding											
12	1	1	41	147	0	1	98	0	10	2	0
21	0	2	55	40	0	1	82	0	20	1	1
9	1	1	31	141	0	2	25	0	10	2	0

**Table 3:** Number of samples for training and testing.

Set	Total Samples	Class 0	Class 1
Training	734	321	413
Testing	184	89	95

**Table 4:** Results using the original feature set.

Model	Accuracy	Class	Precision	Recall	F1 Score
DT	0.77	0	0.75	0.75	0.75
		1	0.79	0.79	0.79
		Avg	0.77	0.77	0.77
ADA	0.83	0	0.80	0.83	0.81
		1	0.85	0.82	0.84
		Avg	0.82	0.83	0.83
SVM	0.86	0	0.85	0.85	0.85
		1	0.87	0.87	0.87
		Avg	0.86	0.86	0.86
RF	0.85	0	0.85	0.81	0.83
		1	0.85	0.88	0.86
		Avg	0.85	0.84	0.85
ETC	0.88	0	0.87	0.86	0.86
		1	0.88	0.89	0.89
		Avg	0.87	0.87	0.87
LR	0.86	0	0.86	0.85	0.85
		1	0.87	0.88	0.88
		Avg	0.86	0.86	0.86
SGDC	0.71	0	0.86	0.43	0.57
		1	0.66	0.94	0.78
		Avg	0.76	0.68	0.67
SGLV	0.87	0	0.85	0.83	0.84
		1	0.86	0.88	0.87
		Avg	0.86	0.86	0.86

**Table 5:** Experiment results using the convolutional feature set.

Model	Accuracy	Class	Precision	Recall	F1 Score
DT	0.79	0	0.78	0.76	0.77
		1	0.80	0.82	0.81
		Avg	0.79	0.79	0.79
ADA	0.88	0	0.85	0.88	0.86
		1	0.90	0.87	0.89
		Avg	0.87	0.88	0.87
SVM	0.88	0	0.85	0.88	0.86
		1	0.90	0.87	0.89
		Avg	0.87	0.88	0.87
RF	0.89	0	0.93	0.82	0.87
		1	0.86	0.95	0.90
		Avg	0.90	0.89	0.89
ETC	0.85	0	0.81	0.85	0.83
		1	0.88	0.84	0.86
		Avg	0.85	0.85	0.85
LR	0.90	0	0.93	0.87	0.90
		1	0.88	0.94	0.91
		Avg	0.90	0.90	0.90
SGDC	0.88	0	0.85	0.86	0.86
		1	0.89	0.88	0.89
		Avg	0.87	0.87	0.87
SGLV	0.92	0	0.93	0.91	0.92
		1	0.92	0.94	0.93
		Avg	0.92	0.92	0.92

**Table 6:** Models’ accuracy using a different number of features.

Model	Features			
	10,000	15,000	20,000	25,000
DT	0.67	0.78	0.79	0.79
ADA	0.80	0.83	0.83	0.88
SVM	0.84	0.85	0.85	0.88
RF	0.86	0.82	0.84	0.89
ETC	0.83	0.81	0.83	0.85
LR	0.86	0.84	0.88	0.90
SGDC	0.86	0.83	0.81	0.88
SGLV	0.86	0.86	0.88	0.92

**Table 7:** Number of correct and wrong predictions for all models.

Model	CNN Features		Original Features	
	CP	WP	CP	WP
DT	146	38	142	42
ADA	161	23	152	32
SVM	161	23	158	26
RF	164	20	156	28
ETC	156	28	161	23
LR	166	18	159	25
SGDC	161	23	130	54
SGLV	170	14	160	24

**Table 8:** Performance validation using four different datasets.

Dataset	Accuracy	
	CNN Features	Original Features
CHD	0.93	0.82
SHD	0.90	0.80
SAHDD	0.77	0.75
HFPD	0.92	0.86

**Table 9:** Architecture of deep learning models.

Model	Hyper-Parameter Setting
GRU	Embedding (1000, 100, 11)
	Dropout (0.2)
	GRU (64, return_sequences = True)
	Dense (2, activation=’softmax’)
	loss = ’binary_crossentropy’, optimizer = ’adam’, epochs = 100
CNN	Embedding (1000, 100, 11)
	Conv1D (128, 2, activation = ’relu’)
	MaxPooling1D (pool_size = 2)
	Flatten ()
	Dense (2, activation = ’softmax’)
	loss = ’binary_crossentropy’, optimizer = ’adam’, epochs = 100

**Table 10:** Results for deep learning models used in the study.

Model	Accuracy	Class	Precision	Recall	F1 Score
CNN	0.82	0	0.77	0.77	0.77
		1	0.85	0.85	0.85
		Avg	0.81	0.81	0.81
GRU	0.81	0	0.75	0.78	0.77
		1	0.85	0.83	0.84
		Avg	0.80	0.80	0.80

**Table 11:** Comparison of computational complexity.

Models	Using Original Features	Using CNN 25,000 Features
DT	0.01	9.45
ADA	0.43	105.01
SVM	1.79	4.99
RF	0.38	7.90
ETC	0.33	15.45
LR	0.01	2.97
SGDC	0.01	0.93
SGLV	7.99	17.06

**Table 12:** Performance comparison with recent studies for heart disease prediction.

Dataset	Ref.	Year	Model Used	Accuracy
CHD	[22]	2019	HRFLM	0.880
	[31]	2021	LR	0.868
	[32]	2021	Naïve Bayes	0.850
	[33]	2021	RF with CDTL	0.893
	[34]	2021	MBAR-CB, CBC	0.918
	Current study	2021	ConvSGLV	**0.930**
SHD	[34]	2021	MBAR-CB, CBC	0.870
	[35]	2021	Ensemble model	0.88
	Current study	2021	ConvSGLV	**0.90**
SAHDD	[34]	2021	MBAR-XGB, XGBC	0.75
	Current study	2021	ConvSGLV	**0.77**
HFPD	Current study	2021	ConvSGLV	**0.92**

## Data Availability

Not applicable.
